# Cell–cell communication in African trypanosomes

**DOI:** 10.1099/mic.0.001388

**Published:** 2023-08-29

**Authors:** K. R. McWilliam

**Affiliations:** ^1^​ Institute for Immunology and Infection Research, School of Biological Sciences, King’s Buildings, University of Edinburgh, Charlotte Auerbach Road, Edinburgh, EH9 3FL, UK

**Keywords:** African trypanosomes, cell–cell communication, coinfection, extracellular vesicles, quorum sensing, social motility

## Abstract

Years of research have shown us that unicellular organisms do not exist entirely in isolation, but rather that they are capable of an altogether far more sociable way of living. Single cells produce, receive and interpret signals, coordinating and changing their behaviour according to the information received. Although this cell–cell communication has long been considered the norm in the bacterial world, an increasing body of knowledge is demonstrating that single-celled eukaryotic parasites also maintain active social lives. This communication can drive parasite development, facilitate the invasion of new niches and, ultimately, influence infection outcome. In this review, I present the evidence for cell–cell communication during the life cycle of the African trypanosomes, from their mammalian hosts to their insect vectors, and reflect on the many remaining unanswered questions in this fascinating field.

## Introduction

Maintaining an active social life is worthwhile if you are a unicellular organism. By sharing information and coordinating their behaviour, unicellular organisms can achieve feats that would not otherwise have been possible if single cells were to have acted alone. For example, cell–cell communication is necessary for the colonization of niches, for appropriately timed cellular differentiation and for efficient division of labour [1, 2]. Single cells produce, receive and interpret signals via a diverse range of mechanisms and communication is not only limited to intraspecies interactions. Indeed, research has presented evidence for interspecies and intergenera communication [3].

African trypanosomes are unicellular eukaryotic parasites that live extracellularly within their broad range of mammalian hosts. In sub-Saharan Africa, *T. brucei rhodesiense* and *T. b. gambiense* are the causative agents of human African trypanosomiasis (HAT), whilst *T. b. brucei*, *T. congolense* and *T. vivax* are responsible for the majority of the animal disease burden. Although the World Health Organization is targeting the elimination of *T. b. gambiense* by 2030, animal African trypanosomiasis (AAT) of domestic animals, particularly cattle, remains responsible for millions of livestock infections and billions of dollars of economic losses annually [4]. African trypanosomes are primarily transmitted cyclically by their tsetse fly vector, although they may also be transmitted mechanically by biting flies [5–8]. The life cycles of trypanosomes are complex, comprising multiple differentiation events within their respective hosts and vectors, and an increasing body of evidence demonstrates that cell–cell communication is an important contributor to parasite pathogenicity and virulence. In this review, I present the current knowledge of cell–cell communication mechanisms during the African trypanosome life cycle and propose potential future areas of study.

## Quorum sensing

Quorum sensing (QS) describes a density-dependant mechanism of cell–cell communication that is widespread in the bacterial world. Bacterial cells release signalling molecules, termed autoinducers, that accumulate in the extracellular environment and are detected by neighbouring cells, allowing the bacteria to monitor the density of their community. Upon reaching a critical cell density, there is a coordinated change in gene expression in the population that, depending of the nature of the signal, elicits a change in the group behaviour [9]. Biofilm formation, bioluminescence and virulence factor production are frequently cited examples of processes regulated by QS [3]. Though commonplace in bacterial systems, QS has also been described in pathogenic fungi and phages, and indeed there is experimental evidence for intergenera and interspecies communication via QS [3, 10].

Upon injection by the tsetse fly, *T. brucei* infections are established in the mammalian host by rapidly proliferating morphologically ‘slender’ forms, which quickly increase in number. Allowed to replicate unchecked, slender parasites would rapidly overwhelm a host, thus limiting the parasite’s transmission potential [11]. As a consequence, it is beneficial that parasites co-operate to regulate their growth *in vivo* to both promote longevity of the host and maximize transmission potential [11]. Such regulation is achieved in *T. brucei* populations in a QS manner, whereby trypanosome cells detect increasing parasite numbers and respond to these cues by activating a developmental switch. During this developmental switch, proliferative slender forms differentiate to ‘stumpy’ forms [12–14]. Stumpy cells are uniformly arrested in G1/G0 [15], and since their abundance increases in response to the first ascending wave of parasitaemia, the parasite population is restricted – prolonging the infection and thus optimizing the transmission of the disease [11]. Beyond the first peak of parasitaemia, stumpy form cells dominate the skin, adipose tissue and bloodstream of chronic-phase rodent experimental infections [11, 16].

In addition to their role in restricting parasitaemia, stumpy cells have conventionally been regarded as the life cycle stage required for transmission. Indeed, differentiation from slender to stumpy cells is accompanied by a host of physiological and gene expression changes that preadapt the cells for tsetse fly uptake and life cycle progression (summarized in [17]). Recently, the requirement of stumpy cells for disease transmission has been challenged [18]. Schuster *et al*. infected teneral tsetse flies with predefined numbers of either replicative slender or cell cycle-arrested stumpy cells (slender and stumpy cells could be distinguished by whether they expressed an NLS-GFP reporter fused to the 3′ UTR of the stumpy-specific marker PAD1 [19]) and found that morphologically slender cells were capable of establishing infections in the midgut of the fly, albeit less successfully than morphologically stumpy cells, even when an infective dose as low as one slender cell/blood meal was used [18]. These slender cells were observed to activate the PAD1 reporter inside the fly and differentiate to the insect procyclic form (PCF) without arresting their cell cycle, prompting Schuster *et al*. to argue that cell cycle-arrested PAD-positive stumpy cells were not required to maintain transmission. Publication of this study has prompted thought-provoking debate surrounding the *T. brucei* life cycle. A full discussion of the arguments for and against the necessity for stumpy cells in disease transmission is outside the scope of this review; however, discussions can be found in [18, 20, 21].

**Fig. 1.**
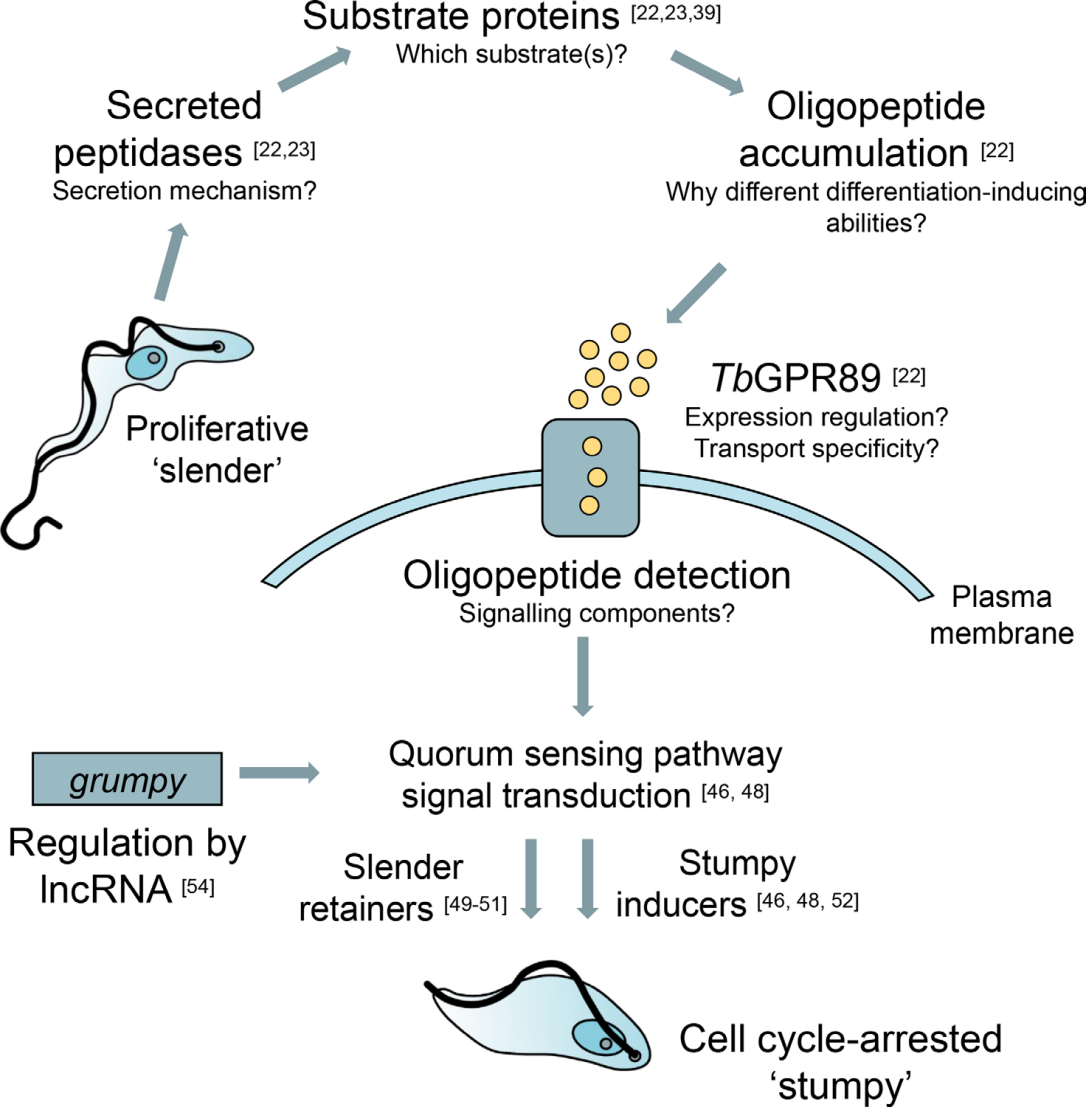
Summary of *T. brucei* slender-to-stumpy differentiation via the QS signalling pathway. Open research questions are posed for the appropriate stages of the QS response. Proliferative ‘slender’ cells secrete peptidases into the extracellular environment via an unknown mechanism. QS-relevant oligopeptides are predominantly generated by two peptidases and accumulate with increasing parasite density. Oligopeptides are transported into the trypanosome via the surface transporter *Tb*GPR89 and begin the QS signalling cascade, resulting in the production of cell cycle-arrested ‘stumpy’ forms. A lncRNA, *grumpy*, contributes towards slender-to-stumpy differentiation regulation and binds at least one member of the QS pathway.

The signal by which *T. brucei* cells communicate parasite density amongst themselves was for decades referred to as an unknown parasite-derived factor termed stumpy induction factor (SIF) that was received by the cells via an unknown receptor [14]. Recent work, however, has now shown that the intercellular QS signal that drives developmental progression from slender to stumpy in *T. brucei* populations is oligopeptides, generated by the proteolytic digestion of host substrates by trypanosome-secreted peptidases (Fig. 1) [22, 23].

Oligopeptides are short chains of amino acid monomers linked by peptide bonds. In a comparison of metabolite levels in fresh and spent bloodstream form (BSF) culture medium, metabolomic analysis found that a number of peptides were slightly enriched in the spent medium, with the most abundant being >10× enriched compared to fresh culture medium [24]. Although this analysis was performed in a cell line incapable of differentiation from the slender to stumpy form, and thus termed ‘monomorphic’, these results are still relevant. Elegant conditioned medium experiments have shown that monomorphic cells still produce the signal that induces differentiation in competent ‘pleomorphic’ cell lines, although they are refractory to their own cues, making them ‘signal blind’ [14]. Indeed, in a metabolomic analysis of the cerebrospinal fluid, plasma and urine of *T. b. gambiense* HAT patients, oligopeptides were significantly enriched in the plasma of infected patients compared to uninfected controls [25]. When pleomorphic parasites were grown *in vitro* in the presence of increasing concentrations of brain heart infusion broth, a source of exogenous oligopeptides, stumpy formation markers, such as increased surface expression of the PAD1 protein and cell cycle arrest, were observed in a concentration-dependent manner [22, 26]. Libraries of synthetic di- and tripeptides were screened *in vitro* for stumpy formation capacity, assessed by PAD1 expression, arrest of population growth and morphology, and it was found that tri-peptides with asparagine, glutamine, histidine, phenylalanine, aspartic acid or tryptophan at their N-termini were the most potent inducers of stumpy formation [22].

The proteolytic digestion of substrates by secreted trypanosome peptidases generates the necessary paracrine signal that communicates increasing parasite density, and accordingly multiple studies have found evidence for the secretion of peptidases by African trypanosomes [23, 27–35]. A number of these secreted peptidases have furthermore been demonstrated to retain catalytic activity in the mammalian host bloodstream. A *T. brucei* serine peptidase, oligopeptidase B, was demonstrated to account for approximately 80 % of total trypsin-like serine peptidase activity in the serum of infected rats but was not detected as being secreted *in vitro* [33], and the type I pyroglutamyl peptidase, a cysteine peptidase, was also demonstrated to hydrolyse the peptide hormones gonadotrophin-releasing hormone and thyrotropin-releasing hormone in the serum of an infected rat, generating an N-terminal tetrapeptide that retained its hormonal activity [35, 36]. Pyroglutamyl peptidase is not apparently actively secreted by trypanosomes during infection, but instead is released by lysed cells [22, 36]. Finally, the activity of a further *T. brucei* serine peptidase, prolyl oligopeptidase, was monitored during the course of a rodent infection. Peaks of prolyl oligopeptidase enzyme activity within the plasma were shown to correlate with observed peaks of parasitaemia within the bloodstream [28]. *In vitro*, prolyl oligopeptidase was shown to hydrolyse purified type I human collagen fibres and peptide hormones [28]. The capability of released pyroglutamyl peptidase and prolyl oligopeptidase to drive stumpy formation was tested by Rojas *et al*. *in vivo* [22]. When mice were infected with pleomorphic cell lines capable of the inducible overexpression of either pyroglutamyl peptidase or prolyl oligopeptidase, induced parasites were found to differentiate to stumpy forms at lower parasitaemia than uninduced cells.

Upon the *in vitro* knockdown of each of the 20 serine peptidases encoded in the *T. brucei* genome, a study found that only 1 of the peptidases, a putative type I signal peptide peptidase, was essential for growth *in vitro* [37]. These phenotypic screens, however, were performed in a monomorphic cell line, incapable of differentiation to the stumpy form. Subsequently, an impressive comprehensive analysis that determined the essentiality and contribution of each secreted *T. brucei* peptidase to QS signal generation in a life cycle competent line of the parasite was performed by Tettey *et al*. [23]. Mass spectrometry identified 12 peptidases secreted *in vitro* by BSF parasites incubated for 2 h in Creek’s minimal medium (a medium that has been developed to contain more physiologically relevant levels of nutrients compared to the standard BSF culture medium [24]). The aforementioned peptidases, pyroglutamyl peptidase and prolyl oligopeptidase, were not among this list of 12, although oligopeptidase B was present. By using a combination of ectopic overexpression, gene knockout and add-back experiments *in vivo*, Tettey *et al*. were able to demonstrate that two of the identified secreted peptidases, oligopeptidase B and metallocarboxypeptidase I, dominate the generation of QS-relevant oligopeptides during infection. Upon overexpression of either of the peptidases *in vivo*, parasites prematurely differentiated to stumpy forms, whereas conversely, when either of the peptidases were knocked out, hypervirulent parasites that exhibited increased parasitaemia and delayed differentiation were generated [23]. Interestingly, a low number of stumpy forms were detected, even when both oligopeptidase B and metallocarboxypeptidase I were deleted in the same cell line, suggesting that a level of redundancy amongst the released peptidases exists. Neither QS-dominant peptidase was predicted to contain an N-terminal sequence for secretion, and in parasites where genetic manipulation had either inactivated the classical secretory pathway or enhanced extracellular vesicle release, the secretion of oligopeptidase B was unaffected. This suggests that, at least in the case of oligopeptidase B, peptidase release is via an unconventional protein secretion mechanism.

*T. brucei* oligopeptidase B has been demonstrated to cleave host atrial natriuretic factor, vasopressin and neurotensin [38], whilst metallocarboxypeptidase I has been proposed to degrade constituent proteins of the blood–brain barrier, such as laminin, collagen and fibronectin [23, 28]. Consistent with this role in degrading matrix proteins, incubation with basement membrane extract, a source of extracellular matrix proteins such as those described above, drives slender-to-stumpy differentiation at low cell density *in vitro* [39].

Environmental oligopeptides, generated by secreted peptidases such as oligopeptidase B and metallocarboxypeptidase I, are transported via a GPR89 family protein, *Tb*GPR89 [22]. *Tb*GPR89 has nine transmembrane domains and is structurally similar to the POT family of oligopeptide transporters, as identified by iTasser homology modelling [40–42]. Interestingly, despite being ubiquitous amongst most prokaryotes and eukaryotes, POT family transporters are missing in African trypanosomes [22]. Functional studies showed that recombinant *Tb*GPR89 could transport a fluorescent dipeptide and thus reproduce the function of conventional POT transporters. *Tb*GPR89 is stage-specifically expressed on the surface of slender-form trypanosomes and cannot be detected in differentiated stumpy forms. Attempts to knockout the gene encoding *Tb*GPR89 were unsuccessful, indicating that *Tb*GPR89 is essential in the bloodstream form of the parasite [22]. Upon ectopic overexpression of *Tb*GPR89, monomorphic parasites refractory to the QS signal displayed only a slight growth defect *in vitro*, whereas developmentally competent pleomorphic cells arrested their growth *in vitro* and *in vivo* in G1, expressed PAD1 on their surface and morphologically transformed to stumpy cells at low parasitaemia. This accelerated stumpy formation upon the overexpression of *Tb*GPR89 was demonstrated to function via the biologically relevant QS signal transduction pathway (discussed below) and was not an overt stress response. It is not yet understood how the expression of *Tb*GPR89 is regulated, although oligopeptides could contribute to regulation, as has been observed for the mammalian intestinal peptide transporter (PepT1) [43, 44]. Whether there is specificity in the oligopeptides transported by *Tb*GPR89, in contrast to general oligopeptide transport, and how this specificity is attained, remains another important unanswered question [45].

Reception of the oligopeptide signal via *Tb*GPR89 stimulates a signal transduction pathway that results in morphological, physiological and transcriptomic changes that promote stumpy formation [22, 46]. Exposure of both pleomorphic and monomorphic cell cultures to the membrane-permeable cAMP analogue 8-(4-chlorophenylthio)-cAMP (8-pCPTcAMP) causes the induction of physiologically relevant differentiation events such as rapid G1/G0 arrest, morphological transformation and mitochondrial elaboration [14, 47]. Exploiting monomorphic cells’ potential to undergo cell cycle arrest and become ‘stumpy-like’ in response to 8-pCPTcAMP [47], a genome-wide RNAi screen in a monomorphic cell line selected for cells that became refractory to 8-pCPTcAMP treatment upon the doxycycline-inducible silencing of a gene-involved in the QS signal transduction pathway [46]. The screen identified approximately 30 proteins that together assembled into a complex signalling pathway operating at distinct levels, from signal transducers [e.g. PP1 (*Tb*927.4.3620/30/40) and the α2 subunit of AMPK (*Tb*927.3.4560)] through to predicted RNA-binding effector molecules [e.g. RBP7 (*Tb*927.10.12100)]. The roles of several genes in driving slender-to-stumpy signal transduction were validated *in vivo* in a pleomorphic cell line capable of differentiation. An extragenic suppression approach to elucidate the hierarchical nature of the signalling pathway established the complex, non-linear nature of the signalling pathway [48]. By the nature of the selection method, only genes suppressing slender-to-stumpy differentiation, so-called ‘stumpy inducers’, were selected in the genome-wide RNAi screen, but a number of genes that act to maintain cells in a slender state, so-called ‘slender retainers’ have also been identified, for example TOR4 [49], RDK1/2 [50] and ZFK [51]. Recently, it was demonstrated that a protein kinase identified in the initial genome-wide RNAi screen, *Tb*DYRK, is a central regulator of QS-dependent differentiation. The kinase inhibits slender retainer proteins such as NOT5 and *Tb*927.4.2750 and activates stumpy inducers such as ZC3H20 [52].

Although the hits from the genome-wide RNAi screen have been verified in a pleomorphic cell line [46], differences in the biology between differentiation competent and defective cells may mean that some molecular components of the QS signal transduction pathway remain unidentified. We know that monomorphic cell lines will not arrest their growth in response to incubation in conditioned medium, whereas the conditioned medium from monomorphic cell lines will induce differentiation in pleomorphic cell lines [14]. Furthermore, upon overexpression of *Tb*GPR89, monomorphic parasites do not arrest in G1/G0, unlike their pleomorphic counterparts [22]. Together, this suggests that monomorphic cell lines, such as those used in the Mony *et al*. screen differ in their ability to transduce, not produce, the QS signal. Genome-wide screens in a life cycle competent cell line would surely identify interesting new components that contribute to parasite communication in the mammalian host.

It has been suggested by Saldivia *et al*. that it is in fact the AMPKα1, and not the AMPKα2 subunit identified by Mony *et al*., that is responsible for the positive regulation of slender-to-stumpy differentiation [53]. Upon treatment of monomorphic *T. brucei* BSF cells with 8-pCPTcAMP, it was shown that AMKPα1 phosphorylation was upregulated, whereas AMPKα2 phosphorylation remained unchanged. Upon AMKPα1 activation, transcripts associated with the stumpy form of the parasite, PAD1 and PAD2, were significantly upregulated, as measured by qRT-PCR. The AMKPα1 phosphorylation state was monitored during an experimental *in vivo* infection of mice. In slender-form parasites no phosphorylation was detected, whereas phosphorylation was strongly upregulated during early differentiation events and maintained until the population had fully differentiated. Differentiation to stumpy forms *in vivo* was reduced upon treatment with an AMPKα1 inhibitor, compound C [53].

Following reception by *Tb*GPR89, it remains unknown how oligopeptides feed into the signalling transduction cascade. Given that 8-pCPTcAMP is cell-permeable, the genes involved in the earliest responses were likely bypassed in the Mony *et al*. screen. The discovery that exogenous sources of oligopeptides could drive slender-to-stumpy differentiation *in vitro* [22, 39] presents another exciting experimental tool that could be used to investigate the earliest responses to oligopeptide detection by *Tb*GPR89.

Our understanding of the many regulatory roles played by non-coding RNAs in gene expression control has greatly developed in recent years and, perhaps unsurprisingly, a *T. brucei* long non-coding RNA (lncRNA), regulator of growth and the stumpy formation (*grumpy*), has been described as being involved in the regulation of slender-to-stumpy differentiation [54]. The *grumpy* gene was identified in a thorough reannotation of the lncRNA gene repertoire of *T. brucei* and was shown to be located upstream of the QS genes *RBP7A* and *RBP7B*. The expression of *grumpy* was significantly upregulated after incubation of pleomorphic cells with pCPT-cAMP, as shown by qRT-PCR. When *grumpy* was overexpressed in transgenic parasites containing GFP fused to the 3′ UTR of PAD1 [19] (fluorescence suggests the presence of intermediate or stumpy cells), GFP expression was significantly higher on days 1 and 2 following induction of overexpression compared to the parental cell line. Furthermore, this increase in fluorescence was accompanied by a restriction in cell growth, accumulation in G1/G0 and morphological transformation. *In vivo*, mice infected with parasites overexpressing *grumpy* did not develop a detectable parasitaemia. The functional ability to drive premature stumpy formation was attributed to *snoGRUMPY*, an 88nt snoRNA encoded by *grumpy* that is produced by splicing of the lncRNA, and it was furthermore shown to bind a transcript encoding a hypothetical protein identified by the Mony *et al.* genome-wide RNAi screen.

## SoMo

Co-ordinated cell motility between single-celled organisms allows populations to colonize niches that would not otherwise be reachable unless in a multicellular state [2]. If we first consider bacteria, such behaviour is absent in typical agitated laboratory suspension culture. However, grow bacteria on a solid surface and a host of collective motility behaviours such as twitching, gliding, rippling and swarming can be observed [2, 55, 56]. Co-ordinated movement as a group can allow pathogens to colonize their host successfully. For example, cell–cell signalling drives swarming of *Pseudomonas aeruginosa *, a behaviour that precedes biofilm formation and allows the opportunistic human pathogen to colonize solid surfaces, such as catheters, rapidly [57, 58].

Following trypanosome ingestion within a tsetse blood meal, differentiation to replicative early PCFs occurs within the endoperitrophic space of the midgut lumen [18]. Early PCFs are characterized by the surface expression of a GPEET and EP procyclin coat [59]. GPEET and EP procyclin are two glycosylphosphatidylinositol (GPI)-anchored proteins that have distinguishing amino acid repeats (Gly–Pro–Glu–Glu–Thr for GPEET procyclin or Glu–Pro for EP procyclin) at their C-termini [60–62]. To establish a mature infection of the midgut successfully, the early PCFs must traverse a chitinous barrier called the peritrophic matrix (PM) and invade the ectoperitrophic space [63]. Following successful passage into the ectoperitrophic space, most early PCFs have differentiated into late PCFs that are positive for EP but negative for GPEET procyclin surface expression [64]. From the ectoperitrophic space, cells migrate to the proventriculus and undergo gross morphological restructuring to become epimastigotes that travel onwards to the salivary glands, where they attach to microvilli. Following extensive proliferation, epimastigotes differentiate to mammalian infective metacyclic trypomastigotes and detach in preparation for delivery to the mammalian host.

In 2010, Kent Hill’s laboratory published the first description of social behaviour in the PCF of *T. brucei*, a phenomenon that has since been termed ‘SoMo’ for social motility [56]. Given that trypanosomes are in intimate contact with the structures within their tsetse fly host, the Hill Lab investigated PCF motility when cultivated on semi-solid agarose plates, rather than in suspension culture [56]. Keen powers of observation and an elegant series of time-lapse and live-video microscopy experiments demonstrated that, when plated on semi-solid agarose, PCF trypanosomes will multiply at the site of inoculation and form projections of hundreds of thousands of cells that begin to migrate in a clockwise direction in a coordinated and polarized fashion [56, 65]. Migration begins once the cell density at the inoculation site surpasses a threshold of 1.6e6 cells 5 µl^−1^ [65] and the projections move forward at a rate of a few microns per minute, equivalent to approximately 500 body lengths a day [56, 66]. Formation of projections was an active process dependent on a functional flagellum, as demonstrated by knockdown of trypanin, a regulator of flagellar motility [56, 67]. Neighbouring projections maintained a constant distance from one another and never crossed paths, with projections instead either stopping or diverting their forward movement to avoid contact [65]. This suggested that the migrating cells were able to sense each other’s presence and, moreover, coordinate their collective movement in response to external cues from neighbouring projections.

**Fig. 2.**
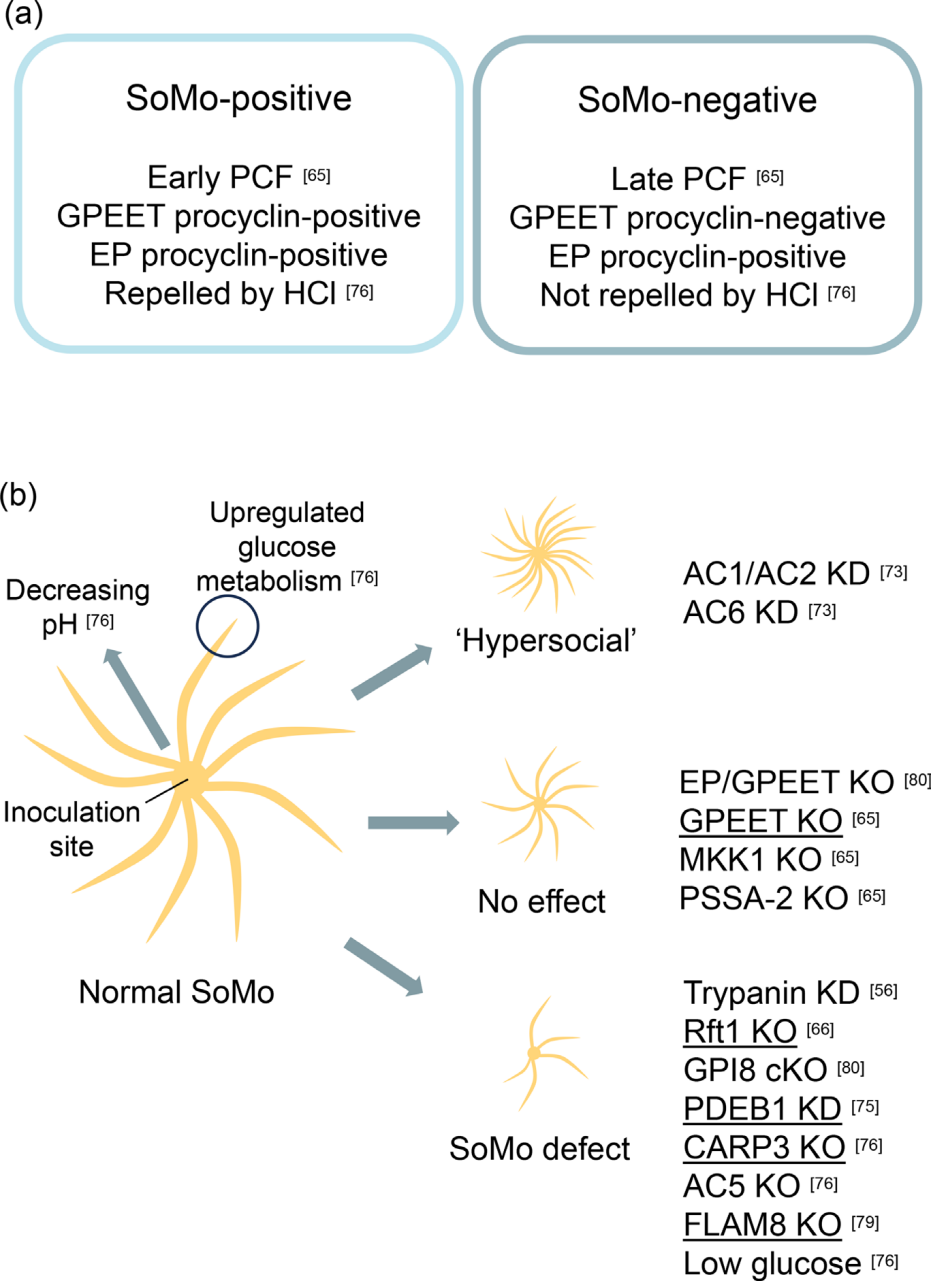
(**a**) Characteristics of SoMo-positive and -negative cells. (**b**) Summary of SoMo phenotypes for *in vitro* and *in vivo* tested conditions. Graphic depicts SoMo migration patterns on semi-solid agar plates. Genes/growth conditions on the right have been validated *in vitro*. Genes that have additionally been tested for their ability to impact on/block parasite migration from the endoperitrophic space to the ectoperitrophic space *in vivo* have been underlined. KD, knockdown; KO, knockout; cKO, conditional knockout.

By assessing the surface coat composition of the cells contained within forward moving projections, Imhof *et al*. demonstrated that SoMo was restricted to EP and GPEET double-positive cells, i.e. early PCFs [65]. Although late PCFs were able to establish colonies at the site of inoculation, only early PCFs began coordinated movement as projections. Furthermore, despite being SoMo-negative, late PCFs still appeared capable of producing a repellent activity, since early PCFs could sense and coordinate their movement away from colonies of late PCFs [65]. As previously mentioned, early PCFs are found in the tsetse fly midgut lumen shortly after a blood meal before migrating to the ectoperitrophic space and then differentiating to GPEET-negative late PCFs. Imhof *et al*. demonstrated that GPEET was not essential for SoMo, drawing a parallel to lack of GPEET essentiality for colonization of the ectoperitrophic space [65, 68]. Thus, putting SoMo into a true biological context, it appeared that SoMo was a property of the life cycle of the parasite responsible for early colonization of the fly midgut (Fig. 2a). Adding weight to this hypothesis, mitogen-activated protein kinase kinase 1 (MKK1) null mutants and procyclic-specific surface antigen 2 (PSSA-2) null mutants (both capable of developing midgut infections but unable to establish salivary gland infections) were still positive for all features of SoMo [65, 69, 70].

Coordinated *in vitro* social motility on agarose plates was first linked to an *in vivo* phenotype 5 years after the initial observation of the collective group behaviour. Early PCF null mutants of Rft1, an endoplasmic reticulum protein implicated in the synthesis of N-linked glycans, needed to reach a threshold cell density within colonies that was approximately twofold higher than that of wild-type (WT) cells to begin outward movement of projections [66]. When projections did form, far fewer were generated than in WT cells. The motility of Rft1 null mutants in culture was unaffected [66]. When the tsetse infection prevalence and intensity was compared between Rft1 null mutants and WT cells, no difference was found on day 3 post-infection (p.i.), indicating that the absence of Rft1 did not affect the ability of early PCFs to survive in the midgut lumen. In contrast, on day 14 p.i., mature ectoperitrophic infections were up to fourfold less likely with Rft1 null mutants than WT cells. The results of this study therefore supported the notion that SoMo is reflective of early PCF migration from the endo- to the ecto-peritrophic space, and suggest that N-linked glycans comprise a component of the machinery that regulates the collective social behaviour [66].

In a fruitful year for SoMo research, it was also discovered that cAMP signalling plays an important regulatory role. Cyclic nucleotides, such as cAMP, regulate a wide range of cell–cell signalling events and are produced from ATP by adenylate cyclases (ACs) [71]. The *T. brucei* genome encodes a large multigene receptor-type AC family, the majority of which localize to the flagellum and, coincidentally, are N-glycosylated [72]. ACs 1–6 are specifically upregulated in the PCF form and localize to the flagellar membrane [72]. RNAi knockdown of either AC1 and AC2 together or AC6 alone created a ‘hypersocial’ phenotype, whereby a greater number of less well-spaced projections were formed from colonies spotted on semi-solid agarose plates [73]. Mutation of the AC6 active site demonstrated that it was the loss of the adenylate cyclase activity and not loss of the entire protein that was responsible for the hypersocial phenotype [73]. Loss of AC activity would trigger a decrease in local cAMP levels. Correspondingly, when an increase of local cAMP levels, as measured by a FRET-based cAMP sensor, was induced by pharmacological inhibition of cAMP-specific phosphodiesterase (PDE) activity, SoMo activity was completely ablated [74]. PDE inhibition did not affect suspension culture motility. *Tb*PDEB1 localizes to the flagellum and its knockdown phenocopied the pharmacological inhibitor, blocking SoMo *in vitro. In vivo*, it was latterly demonstrated that PDEB1 knockout trypanosomes were less able to establish midgut infections than WT cells, and those infections that did establish were of a lower intensity [75]. Whilst WT midgut infections progressed and established heavy proventriculus infections in all 13 flies after 14 days, only 1 of the 10 flies infected with PDEB1 knockout trypanosomes developed a proventriculus infection. Furthermore, when the PM within the midgut was labelled to better distinguish between the lumen and ectoperitrophic space following dissection, PDEB1 null mutants were found to be stuck in the lumen, having been unable to traverse the PM [75].

The above studies suggested that early PCFs probably released a diffusible secreted factor (or factors) into the environment that acted as chemotactic cues to initiate SoMo and repel neighbouring projections. In an impressive study, Shaw *et al*. recently demonstrated that self-generated pH gradients act as mediators of SoMo on semi-solid agarose plates [76]. The pH of semi-solid agarose plates was found to first decrease within the centre of a colony, with the pH of the plate periphery gradually decreasing over the course of a week, creating a gradient. Projections of early PCFs were strongly repelled by exogenous HCl, whilst late PCFs did not respond. Alternatively, both PCF stages were attracted by exogenous NaOH, with even the late PCFs forming projections in its direction. Interestingly, trypanosomes undergoing SoMo have also been demonstrated to be strongly attracted towards actively growing *Escherichia coli *, a behaviour termed ‘BacSoMo’ [77]. Such is the strength of the positive chemotaxis, trypanosome projections that would normally repel one another, move across each other to reach the bacteria [77]. RNA-seq data showed that cells at the tip of SoMo projections upregulate a number of glucose transport and metabolism components compared to those at the base of the projection. PCFs incubated on agarose plates with low glucose concentrations were defective for SoMo (GPEET expression and population doubling time were not negatively impacted) and little change in the environmental pH was detected. In contrast, when cells were grown on plates containing high concentrations of glucose, SoMo was present and the environment was strongly acidified, demonstrating a link between glucose metabolism, pH gradient formation and the ability to perform SoMo [76].

Given (a) the prior identification of cAMP signalling in the regulation of SoMo [75] and (b) the differential expression of several ACs in cells at the tip of SoMo projections compared to the root [76], Shaw *et al*. explored the role of cAMP signalling in pH taxis. PDEB1 null mutants were completely unresponsive to exogenous sources of acid and alkali and knockout of CARP3, a cAMP response protein that mediates resistance to the PDEB1 inhibitor Cpd A [78], similarly abrogated the cell’s response to acid. Pulldown of tagged CARP3 identified AC5, a PCF-specific AC that localizes to the flagellar tip [72], and this was also demonstrated to be essential for the cell’s response to acid. Together, these results demonstrated that the cAMP signalling components PDEB1, AC5 and CARP3 mediate pH taxis and thus, SoMo [76]. In comparison to WT cells, CARP3 null mutants establish fewer midgut infections, and any infections that do establish are of low intensity and do not progress to the proventriculus. The localization of CARP3 is important for its function, since relocalization away from the flagellar tip by deletion of its N-terminus or flagellar member 8 (FLAM8) abolished SoMo and decreased vector infectivity [79]. Furthermore, it has recently been reported that CARP3 is responsible for regulating the expression of multiple AC isoforms, including AC1 and AC6, which have been previously implicated in regulating SoMo behaviour [79].

In addition to the above mentioned SoMo components, it has also been recently described that GPI-linked surface proteins are mediators of SoMo and developmental progression [80]. Using a conditional overexpression of the GPI–transamidase complex subunit *Tb*GPI8 in a null mutant background, it was found that the abolition of processing and transport of GPI-anchored proteins was accompanied by differentiation from early to late PCFs and loss of SoMo *in vitro*. It has been demonstrated that neither EP nor GPEET procyclin are essential for SoMo, and therefore it remains to be seen which GPI-anchored protein/proteins is/are required [65, 80].

Although the same signalling machinery (Fig. 2b) regulates *in vitro* SoMo and *in vivo* migration through the midgut, is the coordinated group movement, seen as projections on semi-solid agarose plates, replicated within the fly? Our only clue thus far comes from a beautiful study of trypanosome morphology and motion by Schuster *et al*. [81]. High-speed fluorescence microscopy showed that PCF cells were capable of movement as individuals within the midgut of the fly. However, when cells encountered a physical barrier that prevented further forward motion, clusters of cells formed that then synchronized their flagellar beats. Within the midgut, such an oscillating cluster of cells could be observed. Synchronization of flagellar motion was not, however, restricted to the life stages that are SoMo competent. Nevertheless, this observation raises a number of fascinating questions. In addition to pH taxis, do other environmental factors, specifically the physical niche and relative confinement, also govern SoMo? How do the trypanosomes detect blockades? How is the coordination of flagellar beating within a group of cells achieved? Migration through the fly does not stop following invasion of the endotrophic space and cells must continue onwards to the salivary glands to be successfully transmitted. Given that the synchronization of flagellar motion in the tsetse fly vector was not limited solely to the PCF [81], this suggests that in other stages of the parasite in insects at least some level of collective motion, and thus cell–cell communication, exists.

## Membranous nanotubes and extracellular vesicles

Membranous nanotubes and extracellular vesicles (EVs) are key mediators of short- and long-distance cell–cell communication in both prokaryotic and eukaryotic systems. Membranous nanotubes are thin protrusions from the plasma membrane that connect cells at a distance of up to 150 µm and are formed by one of two mechanisms – by actin driven protrusions at the plasma membrane or by neighbouring cells coming into contact and then separating [82, 83]. The channels formed by membranous microtubes can transfer calcium fluxes between connected cells, and thus mediate direct cell–cell signalling, or transfer cargo as large as organelles [82, 83]. EVs are cell-derived membranous vesicles that are released into the extracellular environment and carry a diverse range of cargoes, including surface-exposed membrane proteins, genetic material, lipids and cytosolic proteins within their lumen [84]. The umbrella term ‘EV’ comprises a diverse range of membranous vesicle subtypes that each vary in their biogenesis, size, nature and role [84, 85]. Exosomes, for example, are 30–150 nm in size and are formed within the endocytic pathway by inward budding of the endosomal membrane, whereas microvesicles are 100–1000 nm in size and are formed by outward blebbing of the plasma membrane [84, 86]. EVs facilitate signalling between cells by one of three methods: activation of cell surface receptors; fusion with the recipient cell membrane and subsequent delivery of cargo; or endocytosis by the recipient cell [84].

The earliest observations of *T. brucei* membranous nanotubules and EVs were made decades prior to our understanding of their composition, cargo and role in trypanosome cell–cell signalling. In the 1960s, Vickerman and Luckins observed the presence of ‘long thread-like appendages’ coated in variable antigen in *T. b. brucei* that they termed plasmanemes [87], whilst later, in the 1970s, similar filaments were described for *T. b. rhodesiense* during the process of differentiation [88]. Interestingly, electron microscopy identified two different forms of *T. b. rhodesiense* filaments: ‘short thick’ filaments that originated in the Golgi complex and were secreted from the flagellar pocket of stumpy form cells during *in vivo* infections and ‘long thin’ filaments that were cytoplasmic extrusions originating from slender form cells *in vitro* [88]. Various forms of filaments and ‘filopodia’ protrusions have also been observed in *T. congolense in vitro* [89].

*T. brucei* ‘plasmanemes’, now termed membranous nanotubes, were characterized much later in more detail by Szempruch *et al*. [90]. Differential interference contrast microscopy and live imaging with a membrane-binding dye revealed that *in vitro*- and *in vivo*-generated membranous nanotubes from slender form cells are highly dynamic structures that are 2–20 µm in length, develop from the flagellar membrane at the posterior end of the cell and are bound by a lipid membrane. The membranous nanotubes could either form stable interactions between cells over distances up to and beyond 20 µm, or could dynamically branch and probe the extracellular environment, forming multiple connections to the posteriors of neighbouring cells [90]. Given the stable nature of these connections between trypanosomes, and that *in vitro* protrusions were induced by placing the cells under stress, it may be that these membranous nanotubules form active cell–cell links that initiate communication and the active sharing of cellular components that promote the survival of the population under challenging conditions.

**Fig. 3.**
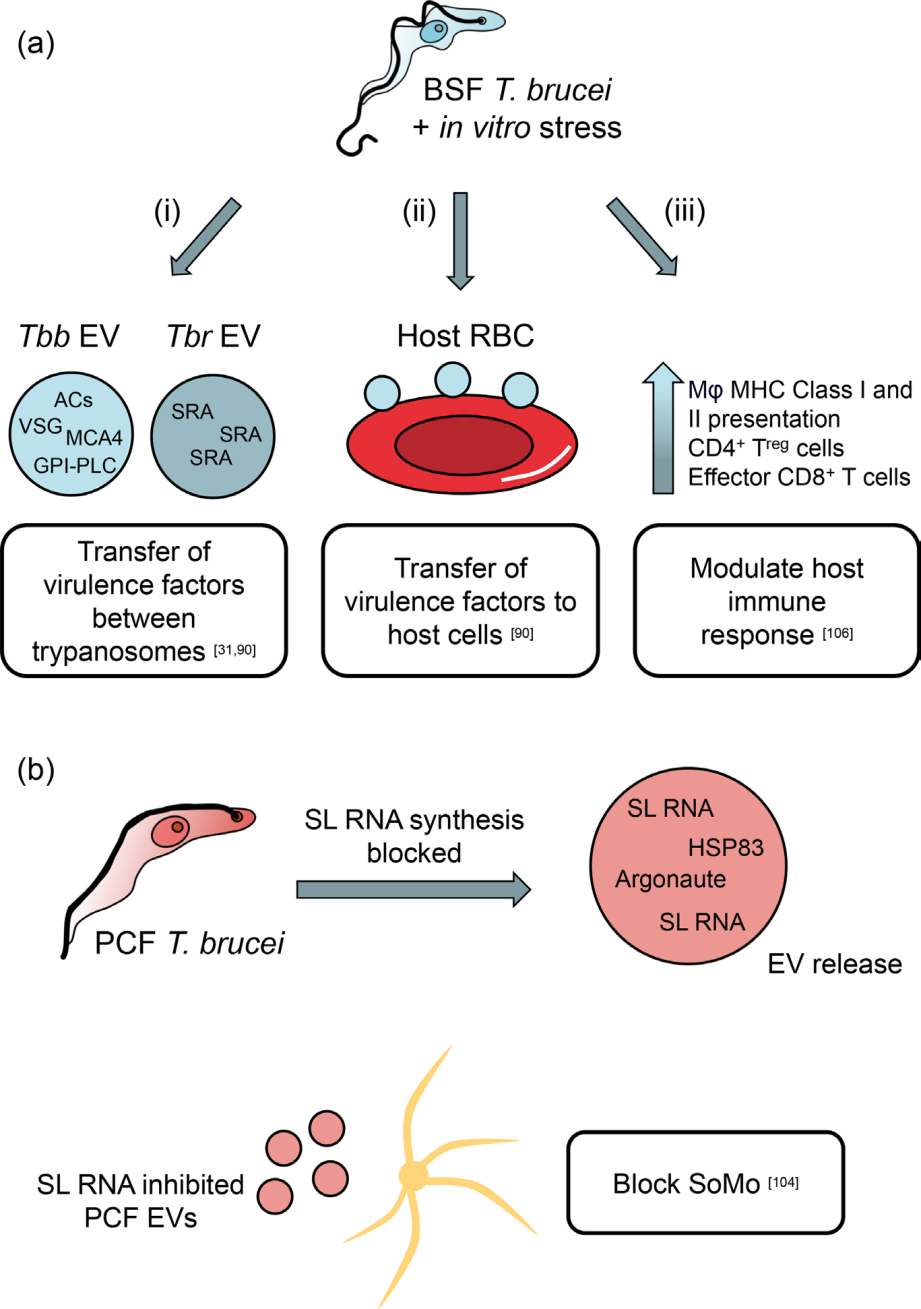
Proposed biological roles for (**a**) BSF and (**b**) PCF *T. brucei* EVs, based on *in vitro* evidence. (**a**) BSF *T. brucei* EVs are proposed to do the following (i–iii). (**i**) Mediate virulence factor transfer between neighbouring trypanosomes. Upon induction of stress *in vitro*, purified *T. b. brucei* EVs were shown to contain multiple flagellar and membrane proteins that were known virulence factors. In addition, *T. b. rhodesiense* (resistant to human serum) EVs were shown to confer human serum resistance to *T. b. brucei* (human serum-susceptible) cells through the transfer of SRA. (ii) Transfer virulence factors to host cells. *T. b. brucei* EVs fused with host RBCs and incorporated VSG protein into the lipid membrane, changing the biophysical properties. (iii) Modulate the host immune response. *T. brucei* EVs established communication with mouse mononuclear cells and influenced both the innate and adaptive arms of immunity. This is proposed to help establish an environment that promotes trypanosome perseverance without overwhelming the host. (**b**) When synthesis of SL RNA was blocked, PCF cells released EVs that were capable of blocking SoMo of WT PCF cells *in vitro*.

Membranous nanotubes vesicularize and dissociate from ‘donor’ cells to form cargo-loaded EVs. Geiger *et al*. used electron microscopy to observe the budding and release of 50–100 nm vesicles originating from the plasma membrane and flagellum of BSF and PCF *T. b. gambiense* parasites [31]. The vesicles were present following both the incubation of parasites in protein-free secretion medium and the isolation of BSF parasites from infected rodent blood. Identification of proteins such as ubiquitin, RAB and clathrin heavy chain, proteins common to most exosomes [31, 91], in their secreted protein dataset supported the authors’ hypothesis that *T. b. gambiense* was releasing macromolecule-containing vesicles into the environment. A comparison of protein profiles by SDS-PAGE found that the contents of sucrose-fractionated vesicles did not differ significantly from the entire secreted proteome [31]. Upon *in vitro* induction of stress against *T. b. brucei* cells, Szempruch *et al*. observed an accumulation of ‘chains’ of vesicles with a diameter of ~100 nm resembling ‘beads on a string’, similar to those visualized by Geiger *et al*., which dissociated to form free diffusible EVs [31, 90]. The proteome of the isolated EVs was found to contain a considerable number of flagellar and plasma membrane proteins and abundant proteins such as VSG (Fig. 3a). Interestingly, a number of the flagellar proteins were important virulence factors in the bloodstream stage of the parasite’s life cycle, such as ACs [71, 73, 76, 79, 92], metacaspase 4 [93] and glycosylphosphatidylinositol phospholipase C [94, 95].

A role for EVs in BSF trypanosome communication was proposed by Szempruch *et al*. [90]. EVs purified from a BSF *T. b. brucei* cell line expressing the serum resistance-associated (SRA) gene of *T. b. rhodesiense*, a virulence factor that confers human infectivity [96, 97], were found to contain the SRA protein. For EVs to mediate intercellular communication via the transfer of macromolecules, the membrane-bound vesicle must first fuse with the target membrane before its encapsulated cargo can be released. *T. b. brucei* EVs that were labelled with a lipophilic fluorophore were shown to fuse with the lipid bilayer of neighbouring *T. b. brucei* cells, with the fluorescent signal initially localizing to the flagellar pocket of the recipient cells before dispersing across the entire surface [90]. Fusion was non-saturable, confirming a lack of receptor-mediated endocytosis. It was furthermore demonstrated that following fusion with the target cell membrane, the EV cargo was delivered and that this could alter the physiology (and in this instance, virulence) of the recipient trypanosome cell. When WT *T. b. brucei* were incubated with *T. b. rhodesiense* EVs, the internalization of EV encapsulated SRA into the endocytic pathway of *T. b. brucei* conferred human serum resistance in the previously non-human infective cell line [90]. Thus, trypanosome EVs were demonstrated to fuse with neighbouring cells and mediate the transfer of virulence factors that would promote survival in the mammalian host (Fig. 3a). Whether this transfer of SRA from *T. b. rhodesiense* cells to non-human infective trypanosomes as a survival strategy is operative in the field, however, remains unanswered. As acknowledged by Szempruch *et al*., although human infections with *T. b. brucei* have been reported [98], these may be the result of misidentification of the infective species or sexual crosses between animal and human infective trypanosomes in the tsetse fly, resulting in the inheritance of the SRA gene [99].

The capability of diffusible EVs to expedite cell–cell communication and modification of behaviour between neighbouring African trypanosome cells is not only relevant in the BSF of the parasites, but has been demonstrated to be effective in the PCF insect form too (Fig. 3b). The spliced leader (SL) is a sequence present at the 5′ of all mature trypanosome mRNAs and is donated from SL RNA during the *trans*-splicing of trypanosome polycistronic mRNA [100–103]. When synthesis of the SL RNA was disrupted in PCF trypanosomes by RNAi of a splicing component or heat shock at 37 °C, SL RNA was exported to the cytoplasm and formed SL RNA granules (distinct from stress granules and P-bodies), which were then secreted by the cell within exosomes that also contained Argonaute and HSP83 [104]. These exosomes, only present in the disrupted cells, were ~100 nm in size, consistent with previous observations, and formed in multivesicular bodies in a process that was dependent on the endosomal sorting complex required for transport (ESCRT) machinery [104]. The perturbation of splicing or the ESCRT machinery did not inhibit the formation of nanotubes similar to those observed by Szempruch *et al*., indicating that there are at least two mechanisms by which EVs can be secreted from cells [90, 104].

As previously discussed, PCF trypanosomes engage in a social behaviour termed SoMo, whereby populations of cells coordinate their movement and migrate as one [56]. When plated on semi-solid agar alongside WT cells, the splicing-inhibited cells were unable to coordinate their movement due to a lack of GPEET procyclin expression. Their presence, however, seemed to act as a SoMo repellent, with WT cells halting their movement towards them sooner than observed between two WT populations plated together. When splicing-inhibited cells that were engineered to create a block in exosome secretion were plated alongside WT cells, the WT cells approached the mutant population as closely as they would another WT population, suggesting that the exosomes were responsible for delivering a signal to neighbouring cells that acted as a repellent [104]. Plating of WT cells with exosomes purified from splicing-inhibited cells supported this hypothesis (Fig. 3b). It thus appears that, at least *in vitro*, upon the induction of stress, PCF trypanosomes will secrete exosomes that actively repel the approach of neighbouring projections of migrating trypanosomes, perhaps conveying the message that their neighbours have a better chance of survival if they actively avoid their current environment. Whether EVs play a role in mediating the migration of parasites *in vivo* remains to be reported. Nevertheless, it would be interesting to investigate the active component, or components, within the exosomes that act as the repellent, the signalling pathways that were activated and the gene expression changes, for example, that were changed as a result of this. Given the current state of knowledge regarding signalling and SoMo, as discussed above, it could be hypothesized that the contents of these vesicles somehow cause changes to the local environmental pH that are detected via the cAMP signalling pathway [74, 76].

EVs released by African trypanosomes not only fuse with and modulate the behaviour of neighbouring trypanosomes, but their contents can furthermore modify the behaviour of their mammalian hosts’ cells (Fig. 3a). *T. b. brucei* EVs, purified from *in vitro* parasites subjected to stress, were able to fuse with and transfer VSG protein to the lipid bilayer of mammalian erythrocytes [90]. Following incubation with EVs, the human erythrocytes displayed modified biophysical properties, with the plasma membrane becoming more rigid and less susceptible to osmotic lysis (though this change was not directly linked to the incorporation of the parasite VSG). When mouse erythrocytes that had been incubated with *T. b. brucei* EVs were injected into naïve mice, they were rapidly cleared from circulation and within an hour of the injection of purified *T. b. brucei* EVs into mice, erythrocyte numbers had decreased by up to 10.6 % [90]. In both human and cattle trypanosomiasis, acute phase of infection is associated with severe anaemia and these results were the first to demonstrate *in vivo* that *T. b. brucei*-derived vesicles could at least be partially responsible for disease morbidity.

Erythrocytes are not the only host cell type that are interacted with during an active *T. brucei* infection. For example, trypanosome infection has been demonstrated to abolish host B cell memory function [105] and ACs (which were coincidentally identified in the proteome of purified *T. b. brucei* EV’s by Szempruch *et al*.) are proposed to subvert the host innate immune response by deactivating the production of TNF-α by first-responder myeloid cells [71, 92]. To specifically investigate the impact of *T. brucei* EVs on the mammalian host innate and adaptive immune system, Dias-Guerreiro *et al*. studied the activation and expansion of mouse macrophage and T cell populations following exposure to either purified *Tb*EVs or co-culture with live trypanosomes [106]. Following 24 h incubation with *Tb*EVs, there was a significant increase in macrophage surface expression of MHC class I and class II molecules, expansion of regulatory CD4^+^ T cells and expansion of effector CD8^+^ T cells. In contrast, incubation with live *T. brucei* cells seemed to have to opposite effect, diminishing the expansion of MHCI^+^, MHCII^+^ and MHC1^+^MHCII^+^ macrophage, and thus minimizing presentation of parasite antigen, and reducing the numbers of CD3^+^CD4^+^ and CD3^+^CD8^+^ T cells [106]. The authors postulate that the opposing effects of *Tb*EVs and trypanosome parasites on innate and adaptive immune cells work in concert to create an environment in which the trypanosomes can inhibit the initial immune response enough to gain a foothold in the host, but not to inhibit the response so much as to overwhelm their host [106].

All of the above studies of nanotubule formation and EVs have been performed *in vitro*, and the *in vivo* validation of a role in cell–cell communication has not been achieved to date. *In vivo* observation of EV production has only been observed once, in scanning electron microscope images of ‘nests’ of *T. brucei* in the lung tissue of infected rodents [107], although the role of these EVs was not elucidated. EV and nanotubule secretions that have been used for *in vitro* functional studies have all been produced under one common circumstance – stress. Whether these extracellular vesicles play a true physiological role in cellular communication *in vivo*, or whether the release of proteins in EVs is simply a response to stress that is designed to overwhelm the immune system (as proposed in [31]) remains to be seen.

If EVs and nanotubules do indeed play a physiological role *in vivo*, it is not difficult to speculate on a number of, as yet untested, scenarios in which they could mediate cell–cell communication. For example, are nanotubules also produced within the ‘nests’ of trypanosomes in extravascular locations, given the close cell proximity? Could extravascular vesicles in mammalian infections communicate a signal that triggers invasion of extravascular tissues or, indeed, reinvasion of the blood? *In vitro* studies have suggested a role for extracellular secreted vesicles in communicating with and manipulating the mammalian host immune system. Could EVs secreted by the life forms found in the insect vector also play a similar role?

## Protein exchange via direct cell–cell contact

Nanotubes and EVs facilitate the intercellular exchange of material between nonadjacent cells. Adjacent cells that are in direct contact with one another may also communicate amongst themselves via the exchange of intracellular material and a number of mechanisms to do so have evolved across the phylogenetic kingdoms. In multicellular animals, gap junctions are protein channels that physically connect the cytoplasm of neighbouring cells and transfer a diverse range of small diffusible molecules, including secondary messengers [108]. Plasmodesmata are cytoplasmic channels that bridge adjacent plant cells and also transfer diffusible signalling molecules [109, 110]. Larger molecules, such as proteins and lipids, are likewise exchanged between cells and myxobacteria, for example, using transient outer membrane fusion to do so [111, 112]. Direct protein exchange between trypanosomes in intimate contact has been investigated by Imhof *et al*. [113].

Upon mixing of GFP or DsRED expressing PCFs in tsetse flies, Imhof *et al*. observed that a small proportion of the population were both GFP- and DsRED-positive, a phenomenon that could be reproduced *in vitro* upon placing the PCFs into fresh culture medium with fresh foetal bovine serum. The proportion of double-positive cells *in vitro* increased upon the deletion of procyclin and in a calcium-dependent manner [113]. Time-lapse and transmission electron microscopy visualized interacting pairs of cells, with intimate contact being made along the flagellum (up to the entire length of the flagellum could be fused), which could be either transient, or stable for several hours. Transfer of soluble cytoplasmic and plasma membrane proteins occurred rapidly and bidirectionally between the pairs, with the flagellum acting as the chief site of protein exchange and distribution. No genetic exchange or cellular fusion was ever observed between the paired cells [113].

As discussed by Imhof *et al*., direct protein exchange could be used *in vivo* to exchange nutrients or communicate between cells, but, given the transient nature of the interaction and the difficulty of distinguishing between mating and protein exchange *in vivo*, any further role for protein exchange in cell–cell signalling between African trypanosomes has not yet been validated [113]. To the best of the author’s knowledge, flagellar fusion and direct protein exchange has not (yet) been reported in any other life cycle stage.

## Coinfection

In most of the above instances of trypanosome–trypanosome communication, the interaction has always been documented between members of the same trypanosome strain/species. In reality however, a population of trypanosomes may not exist in isolation throughout its life cycle [114, 115]. Multiple species of African trypanosome with overlapping host ranges and a shared insect vector co-circulate in the field, presenting ample opportunity for interspecies communication at each stage of the parasite’s life cycle. Indeed, *T. congolense*, *T. brucei* and *T. vivax* coinfections are frequently reported in both the mammalian host and tsetse fly vector [114–121]. Direct and indirect interactions between coinfecting trypanosome species can have significant consequences for the host, infection outcome and the parasites themselves.

**Fig. 4.**
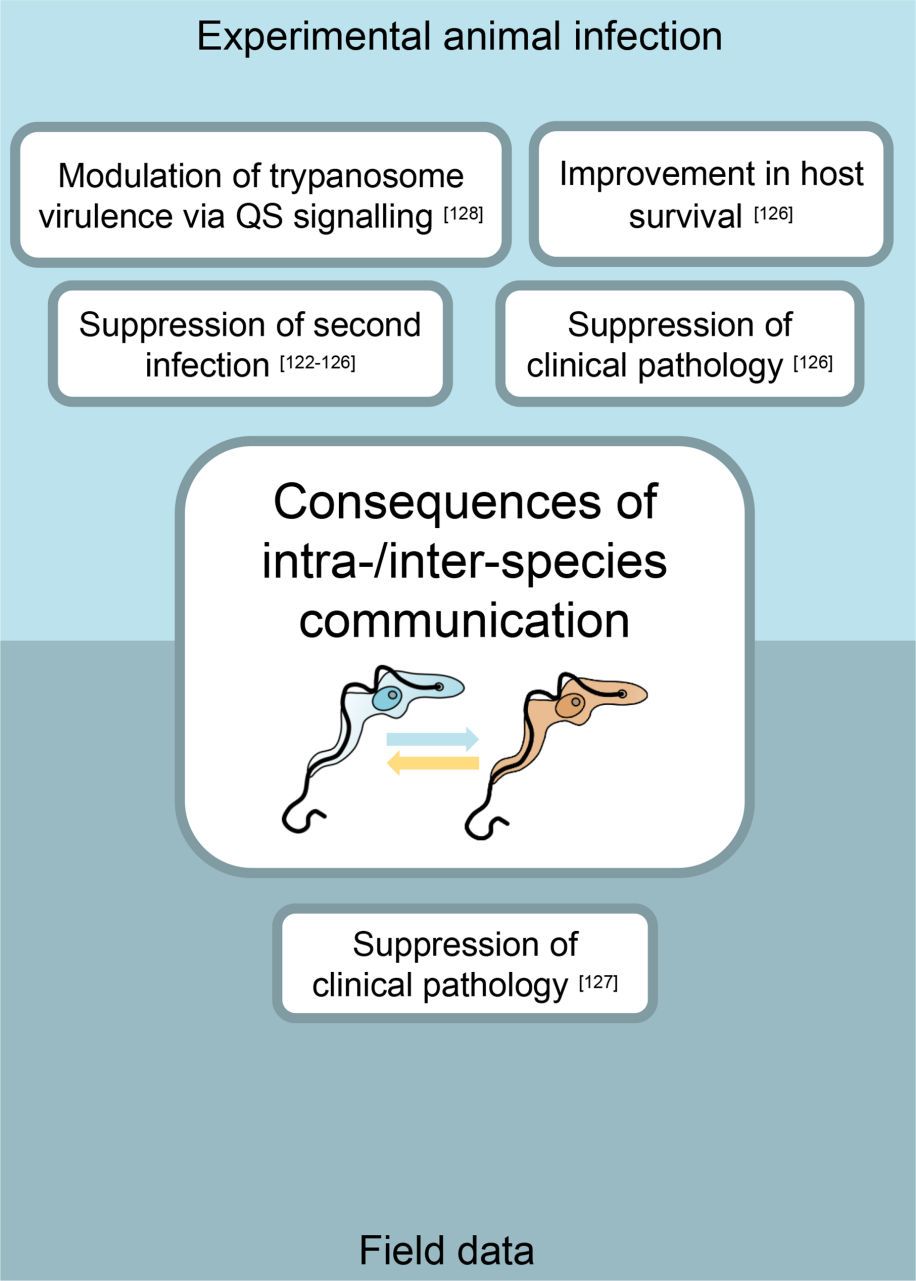
The experimentally validated consequences (for the host and the trypanosome) of intra- and interspecies communication during infection. The consequences in the top panel have been validated in experimental animal infections. The consequences in the bottom panel have been validated by field data.

The impact of intra- and interspecies trypanosome coinfections has been studied in experimental animal model infections (Fig. 4). Ongoing infection with *T. congolense* is observed to suppress a second challenge with an unrelated *T. congolense* strain in goats [122], cattle [123] and rabbits [124]. This suppression was independent of whether the second infection was initiated with infectious metacyclics or proliferative bloodstream forms and the presence of detectable anti-trypanosome antibody [123], suggesting that the phenomenon could be due to either indirect or direct communication between the coinfecting strains, although innate immunity was not ruled out. Ongoing *T. congolense* infection was also able to suppress a secondary *T. brucei* superinfection, as assessed by parasitaemia and the appearance of neutralizing antibodies in the blood or a skin reaction [125]. In contrast, superinfection with *T. vivax* was not suppressed in animals already infected with *T. congolense* [125]. In mice, different strains of fluorescently labelled trypanosomes could competitively suppress one another’s parasitaemia in the early stages of infection [126]. Coinfection with a less virulent strain of *T. brucei* could suppress the parasitaemia of a more virulent strain by up to a third and thus significantly improve host survival. The level of suppression was dependent on both parasite and host factors – the density of the superinfecting strain and the physical condition of the host, respectively [126].

In addition to experimental animal infections, there is also evidence for interaction between coinfecting strains of African trypanosomes in the field (Fig. 4). Whole-genome amplification and subsequent PCR amplification identified *T. brucei*, *T. congolense* and *T. vivax* coinfections in 43 % of 241 working horses and donkeys surveyed in The Gambia [127]. Animals that were infected with either *T. brucei* or, in particular, *T. congolense* alone showed significantly reduced packed cell volume (PCV). However, animals that were infected concurrently with *T. vivax* and *T. congolense* were alleviated of this impact on PCV, suggesting that interactions between coinfecting species of trypanosomes in the field may also result in suppression of virulence and thus clinical pathology [127].

The mechanism and consequences of interspecies communication between *T. brucei* and *T. congolense* have been explored in a laboratory setting. As previously discussed, *T. brucei* undergoes a density-dependent developmental transition from proliferative slender form cells to cell cycle-arrested and morphologically stumpy forms. Although *T. congolense* does not generate morphologically distinct forms, at high parasitaemia the cells also accumulate in G1 [128]. Furthermore, *T. congolense* orthologues of a number of the *T. brucei* QS signalling pathway components were identified, and one of these was demonstrated to functionally complement the loss of its *T. brucei* counterpart *in vivo*, demonstrating the conservation of the QS signalling pathway.

*T. brucei* uses a quorum-sensing response to communicate a differentiation signal between cells. Given the conservation of many QS signalling components, the ability of the *T. brucei* and *T. congolense* cells to ‘talk’ to one another and the consequences of this for parasite virulence were explored in a rodent infection model. In the presence of an established *T. congolense* infection, *T. brucei* were found to arrest their growth and transform to stumpy forms at a lower cell density than in monoinfections [128]. When a member of the *T. brucei* QS signalling pathway was silenced, *T. brucei* cells in a coinfection did not exhibit accelerated stumpy formation, demonstrating that *T. congolense* and *T. brucei* can ‘converse’ *in vivo* via QS and that this cross-talk can modulate the virulence of the coinfecting *T. brucei* strain.

Cross-talk between coinfecting trypanosome species, and subsequent modification of virulence could have important consequences for infection outcomes in the field. Upon transmission to an environment that immediately promotes differentiation, coinfecting trypanosomes that are less sensitive to the QS signal, and thus more virulent overall, may be selected for [128]. In the field, chronic infections of cattle are not uncommon, however, suggesting that rather than selecting for highly virulent trypanosomes, coinfection might actually be important for regulating total parasitaemia, preventing an overwhelming surge of parasitaemia every time an animal is reinfected with infective metacyclics. Alternatively, newly coinfecting trypanosomes could adopt a strategy whereby they ‘hide’ from the resident trypanosome populations by occupying different niches [114, 115]. Indeed, *T. brucei* cells are prolific in the extravascular tissues [129].

Differentiation is only one of many biological events occurring during the African trypanosome life cycle and many more signals may be communicated between coinfecting trypanosomes. Signals communicated between coinfecting species may be for the benefit of the parasite, or to its detriment. Indeed, I have only focused on coinfection of the mammalian host above and perhaps there is also a role for interspecies communication in the insect fly vector. Do other species of African trypanosome undergo coordinated social motility within the fly? And, if so, do the signals produced by some strains or species have a stronger repellent effect on the progression of competing populations of trypanosomes than others? Alternatively, could the pH of the fly midgut be influenced by a pre-existing infection in such a way that it accelerates the progression through the fly of a newly ingested trypanosome population?

## Conclusions

In this review, I have attempted to cover the field’s current state of knowledge regarding cell–cell communication between African trypanosomes. I have furthermore included examples of trypanosome interactions with mammalian host cells, such as erythrocytes, but to cover all of these was beyond the scope of this review, as was discussion of the various proposed machineries and mechanisms responsible for the release of extracellular vesicles.

Once thought of as a phenomenon restricted to the bacterial world, there is ample evidence that cell–cell communication and cooperation play a pivotal role not only in the African trypanosomes’ life cycle, whether that be within the mammalian host or insect vector, but also in various other single-celled parasitic organisms, such as *Leishmania* spp., *Trichomonas* spp. and *Plasmodium* spp. [130]. Given the apparent importance of cell–cell communication for parasites to properly complete their life cycles, improving our understanding of the mechanisms by which these processes occur may provide an opportunity for the discovery of novel biology and the design of novel therapeutic or transmission-blocking interventions.
